# β-Casomorphin-7 as a Potential Inflammatory Marker: How β-Casomorphin-7 Induces Endothelial Dysfunction in HUVEC/TERT2 Cell Lines

**DOI:** 10.3390/biomedicines13112712

**Published:** 2025-11-05

**Authors:** Judit Rita Homoki, Emese Szilágyi-Tolnai, Ildikó Kovács-Forgács, Georgina Pesti-Asbóth, Arnold Markovics, Attila Biró, Péter Dávid, János Lukács, László Stündl, Judit Remenyik, Attila Péter Kiss

**Affiliations:** 1 Center for Complex Systems and Microbiome Innovations, Faculty of Agricultural and Food Sciences and Environmental Management, University of Debrecen, H-4032 Debrecen, Hungary; forgacs.ildiko@agr.unideb.hu (I.K.-F.); georgina.asboth@agr.unideb.hu (G.P.-A.); arnoldmarkovich@gmail.com (A.M.); attila.biro88@gmail.com (A.B.); david.peter@agr.unideb.hu (P.D.); remenyik@agr.unideb.hu (J.R.); 2 Clinical Centre, Health Care Service Units, Clinics, Department of Obstetrics and Gynaecology, University of Debrecen, H-4032 Debrecen, Hungary; lukacs.janos@med.unideb.hu; 3 Institute of Food Technology, Faculty of Agricultural and Food Sciences and Environmental Management, University of Debrecen, H-4032 Debrecen, Hungary; stundl@agr.unideb.hu; 4 Agricultural and Food Research Centre, Széchenyi István University, Egyetem Square 1, H-9026 Győr, Hungary

**Keywords:** β-casomorphin-7, HUVEC, viability, inflammation, endothelial cell homeostasis, vascular physiology, endothelial dysfunction

## Abstract

**Background/Objectives**: Endothelial dysfunction plays a central role in the development of cardiovascular diseases. β-Casomorphin-7 (BCM-7), a biologically active peptide generated during the digestion of A1 β-casein, is presumed to contribute to this process; however, its direct effects on endothelial cells have not been previously investigated. Here, we aimed to assess whether BCM-7 treatment induces endothelial cell dysfunction through inflammatory cytokines and reactive oxygen species (ROS). **Methods**: In our study, we analyzed the effects of BCM-7 (5 µg/mL) in combination with lipopolysaccharide (LPS, 100 ng/mL) on human umbilical vein endothelial cells (HUVECs/TERT2). The cell viability, apoptosis, necrosis, and intracellular reactive oxygen species were measured. Furthermore, proinflammatory cytokines and enzymes involved in the regulation of inflammation were assessed with quantitative real-time PCR. The gene and protein expression of enzymes that regulate inflammation and vascular function, thus maintaining endothelial homeostasis were assessed. **Results**: BCM-7 enhanced intracellular ROS production *p* ≤ 0.001, increased the expression of interleukin-6 (IL-6) and interleukin-8 (IL-8) *p* ≤ 0.001, and was more effective when used in combination with LPS *p* ≤ 0.001. It decreased the expression of cyclooxygenase-1 (COX-1) *p* ≤ 0.05, during 4 h of exposure, whereas it increased the expression of cyclooxygenase-2 (COX-2) *p* ≤ 0.001, lipoxygenase-5 (LOX-5) *p* ≤ 0.01, and nitric oxide synthase 3 (NOS3) *p* ≤ 0.001; prostaglandin D2 synthase (PTGDS) (*p* ≤ 0.05), expression was also increased after short treatment. **Conclusions**: Our results suggest that BCM-7 may contribute to the development of endothelial dysfunction, especially in the presence of LPS, by enhancing oxidative stress and inflammatory response.

## 1. Introduction

Milk contains carbohydrates, fats, protein (32 g/L), and several minerals, including calcium, phosphorus, magnesium, and various trace elements such as zinc and iodine. It is also an important source of vitamins, such as B_2_, B_12_, D, and A [1,2]. Compared to meat, eggs, or legumes, milk is easily digestible and has a well-balanced amino acid composition, making it an essential part of the diet in many cultures [3]. It plays an important role in the human diet from birth and the consumption of cow’s milk/dairy products usually begins very early. When breast milk is not available, formula is the most widely accepted alternative for infant feeding, as animal milk is one of the most common sources of protein [4]. Complementary feeding typically begins at around the sixth month, when formula is used as a complementary source of protein to breast milk [5].

The main protein components of milk include caseins (α-, β-, and κ-casein), which account for approximately 80% of the total protein content, as well as α-lactalbumin, β-lactoglobulin, serum albumin, and immunoglobulins. Caseins are broken down by various enzymes in the gastrointestinal tract, leading to the formation of numerous bioactive peptides, including antimicrobial, antioxidant, immunomodulatory, opioid, mineral transport, and vascular compounds [6], which may contribute to health [7]. β-casein (β-CN) accounts for approximately 30–45% of the casein content of milk [8,9]. Several genetic variants are known, the most common of which are A1 and A2. A2 β-casein is considered to be the ancient, original variant, while A1 developed during breeding; accordingly, ancient Asian and African cattle produce milk containing only A2 β-casein. This difference directly affects the digestibility of the protein in the intestinal tract [10,11,12]. Studies comparing the consumption of A1 and A2 β-casein suggest that A2 milk may have more beneficial health effects [13]. During the digestion of dairy products, β-casein peptides are formed, including β-casomorphin-7 (BCM-7), the amount of which is significantly higher in A1 milk because histidine promotes its formation [14]. The Ile-Pro peptide bond in A2-β-casein creates a “resistant” cleavage site for peptidases. The cyclic structure of proline and the space-filling, hydrophobic side chain of isoleucine make it difficult for the bond to fit into the active site of peptidases. Therefore, BCM-7 is produced in negligible amounts from this variant [13]. In contrast, during the digestion of A1-β-casein—under the action of pepsin, pancreatic elastase, and leucine aminopeptidase—significant amounts of BCM-7 are formed in vitro [15,16], suggesting that complex degradation mechanisms in the human digestive system are required for the release of these peptides. β-casomorphins are capable of binding to opioid μ-receptors (MOR), which are found in the central nervous system, the immune system, and the gastrointestinal tract. As a result, they can influence stress responses, pain perception, and food intake regulation, but under certain circumstances they can also contribute to pathological processes [17,18,19,20,21].

The overproduction of reactive oxygen species (ROS) and the depletion of antioxidant defense systems play a central role in the development of endothelial dysfunction. An imbalance between ROS and antioxidants increases the production of proinflammatory cytokines (e.g., TNF-α, IL-6, IL-8) and the expression of adhesion molecules, which are key to the progression of atherosclerotic processes. GSH is one of the most important cell-protective antioxidants, and its intracellular presence is essential for protecting endothelial cells against oxidative damage, as it contributes to the neutralization of reactive oxygen species and thus inhibits the development of endothelial dysfunction. According to previous research conducted on intestinal epithelial cells, BCM-7 inhibits cysteine uptake [22], thereby reducing the intracellular synthesis of glutathione (GSH) [22,23,24]. A lack or low concentration of GSH leads to an imbalance in the antioxidant system, which causes the production of proinflammatory cytokines TNF-α, IL-6, and IL-8, leading to a deterioration in the functional integrity of the endothelium. Due to these effects of BCM-7, it can be assumed that they may lead to the development of inflammatory processes in endothelial cells. Inflammatory cytokines increase the expression of adhesion molecules, leading to endothelial dysfunction and leukocyte migration [25]. Damage to the glycocalyx and cell junction complexes (tight, adherens, and gap junctions) leads to a weakening of endothelial barrier function. As a result, LDL accumulates in the subendothelial space, triggering atherosclerosis and increasing the risk of coronary artery disease [26].

Numerous animal studies have examined the relationship between β-casein consumption and hypercholesterolemia and atherosclerosis [27]; for example, rabbits consuming A1 milk showed higher cholesterol levels, fatty tissue lesions extending to the aortic arch, and a higher intima/media ratio than animals consuming milk containing A2 β-casein [28]. In human studies, only epidemiological data are available, linking A1-β-casein consumption to an increased risk of several diseases [14]. Experimental results suggest that BCM-7 released from A1-β-casein may promote LDL oxidation [29,30], which can lead to increased circulating serum lipid concentrations. This process is an early risk factor for the development of heart disease and atherosclerosis [30].

The aim of our study was to investigate the extent to which β-casomorphin-7 (BCM-7) can influence the function of endothelial cells. We paid particular attention to how BCM-7 can induce oxidative stress, activate inflammatory processes, and influence the homeostasis of endothelial cells. We also investigated the mechanisms through which it may contribute to the development of endothelial dysfunction. Since the mechanism of BCM-7’s effect on endothelial cell homeostasis has not been fully understood, the aim of our research was to fill this gap and be the first to characterize the effect of this protein fraction on endothelial dysfunction.

## 2. Materials and Methods

β-Casomorphin 1-7 (Sigma-Aldrich, Darmstadt, Germany). All reagents were obtained from the distributor of iBioTech Hungary Ltd. (Budapest, Hungary) and Nucleotest Bio Ltd. (Budapest, Hungary).

### 2.1. Cell Culture Conditions

In the experiments we used immortalized human umbilical cord vein endothelial cells (HUVECs/TERT2) obtained from ATCC (Manassas, VA, USA). Cells were cultured according to the manufacturer’s instructions. HUVECs were grown using M199 medium supplemented (iBioTech Hungary Ltd., Budapest, Hungary) with 10% heat-inactivated Fetal Bovine Serum (FBS) (iBioTech Hungary Ltd., Budapest, Hungary), 1% penicillin/streptomycin (iBioTech Hungary Ltd., Budapest, Hungary), 1% amphotericin B (iBioTech Hungary Ltd., Budapest, Hungary), 2 mM glutamine (iBioTech Hungary Ltd., Budapest, Hungary), and Endothelial Cell Growth Medium-2 (EGM-2) (iBioTech Hungary Ltd., Budapest, Hungary) at 37 °C in a humidified incubator under 5% CO_2_. Media was changed every 48 h until cells reached 80% to 90% confluency. At confluency, cells were either subcultured or used for experiments. For all experiments, cells were grown at passage 1–4. Media as described above was used as control.

### 2.2. Determination of Cell Viability

Cell viability was monitored using the MTT assay, which measures the formation of formazan crystals from tetrazolium salts by mitochondrial dehydrogenases. Cells were plated in 96-well plates at a density of 20,000 cells/well. When the cells reached confluence, we treated them with different concentrations of β-casomorphin 1-7 (0.1, 0.5, 1.0, 5.0, 10.0, 50.0, 100.0 µg/mL) for 24 and 48 h. The cells were then incubated with 0.5 mg/mL MTT solution for 3 h. Formazan crystals were formed in proportion to the number of viable cells. After the formed crystals were dissolved in 100 µL/well of solubilizing solution (81% (*v*/*v*) isopropyl alcohol (Serva, Heidelberg, Germany), 10% (*v*/*v*) Triton X-100 (Serva, Heidelberg, Germany), and 9% (*v*/*v*) 1 M hydrochloric acid (HCl) (Serva, Heidelberg, Germany)). The absorbance was measured colorimetrically at 465 nm using a Clariostar microplate reader (BMG Labtech, Ortenberg, Germany). The results were expressed relative to 100% of the control group.

### 2.3. Determination of Apoptosis

A decrease in mitochondrial membrane potential is one of the earliest markers of apoptosis. Therefore, to estimate the process, the mitochondrial membrane potential of HUVECs was measured using DilC1(5) stain. Cells were seeded in 96-well black plates at a density of 20,000 cells per well. When the cells reached confluence, we treated them with different concentrations of β-casomorphin 1-7 (0.1, 0.5, 1.0, 5.0, 10.0, 50.0, 100.0 µg/mL) for 24 and 48 h. After the removal of supernatants, the cells were incubated for 30 min with DilC1(5) working solution (50 µL/well). After incubation, the cells were washed twice with PBS and the fluorescence of DilC1(5) was measured at 630 nm excitation and 670 nm emission wavelengths using a Clariostar microplate reader (BMG Labtech, Ortenberg, Germany). Results were expressed relative to 100% of the control group. One of the early signs of apoptotic processes is the reduction in the mitochondrial membrane potential. The fluorescence of cyanin dyes, such as DilC1(5) (1,1′,3,3,3′,3′-Hexamethylindodicarbocyanine iodide) is greatly enhanced when incorporated into membranes or bound to lipophilic biomolecules such as proteins, although they are weakly fluorescent in water. This dye diffuses laterally within the cellular plasma membranes, resulting in even staining of the entire cell at their optimal concentrations.

### 2.4. Determination of Necrosis

Necrotic processes were evaluated by SYTOX Green staining. The dye is able to penetrate only necrotic cells with ruptured plasma membranes and then binds to the nucleic acids, whereas intact cells with unimpaired surface membranes show a negligible SYTOX Green staining intensity. Cells were seeded to 96-well black plates at a density of 20,000 cells per well. When the cells reached confluence, we treated them with β-casomorphin 1-7 of different concentrations (0.1, 0.5, 1.0, 5.0, 10.0, 50.0, 100.0 µg/mL) for 24 and 48 h. After the removal of the medium, the cells were incubated for 30 min with 50 µL/well SYTOX Green dye (1 µM dissolved in Dulbecco’s modified Eagle’s medium) and then washed with PBS. The fluorescence of SYTOX Green was measured at 490 nm excitation and 520 nm emission wavelengths by using a Clariostar microplate reader (BMG Labtech, Ortenberg, Germany). The results were expressed relative to 100% of the control group. SYTOX Green fluorescent dye allows the quick determination of cell viability or necrosis. It will not cross intact membranes but will easily penetrate the compromised membranes characteristic of dead cells. The exceptional brightness of the signal produced in dead cells makes SYTOX Green particularly useful for determining the viability of mammalian cells.

### 2.5. Measurement of ROS

Cells were treated in 96-well black plates at a density of 20,000 cells/well. When the cells reached confluence, we treated them with different concentrations of β-casomorphin 1-7 (0.01, 0.1, 0.5, 1.0, 5.0, 20.0, 100.0 µg/mL) for 1 h. Subsequently, they were exposed to 100 µM 2′,7′-dichlorofluorescin diacetate (DCFDA) for 30 min at 37 °C to label intracellular ROS. After incubation, the cells were washed twice with PBS. Thereafter, the labeled cells were monitored every 2 h. Fluorescence intensity (excitation = 485 nm; emission = 530 nm) was measured using a microplate reader (Clariostar; BMG Labtech, Ortenberg, Germany). Results were expressed relative to 100% of the control group.

### 2.6. PCR

Q-PCR was performed on a Roche LightCycler 480 System (Roche, Basel, Switzerland) using the 5′ nuclease assay. Total RNA was isolated by Promega Sv total RNA isolation system (Madison, WI, USA) according to the manufacturer’s protocol. cDNA was reverse transcribed from 1 µg of total RNA using LunaScript RT SuperMix Kit (PCR Biosystems, London, UK). The reaction was implemented using the Luna Universal Probe Q-PCR Master Mix (PCR Biosystems, London, UK) and TaqMan primers and probes (PCR Biosystems, London, UK). As an internal control, glyceraldehyde-3-phosphate dehydrogenase (GAPDH) was used. The amount of transcripts was normalized to those of the housekeeping gene (GAPDH) using the ΔCT method. Finally, relative gene expressions were calculated with the comparative ΔΔCt method according to Livak’s formula.

### 2.7. Statistics

Analyses were performed using Excel (Microsoft Corporation Redmond, WA, USA) and GraphPad Prism Version 8.0 (GraphPad Software, La Jolla, CA, USA) software. In the statistical analysis, ANOVA followed by Bonferroni’s post hoc test was used for the comparison. The results were expressed as mean ± standard deviation. Differences were considered to be statistically significant at *p* < 0.05. Significance was indicated as *, *p* < 0.05, **, *p* < 0.01, and ***, *p* < 0.001 compared to untreated control cells.

## 3. Results

### 3.1. BCM-7 Effects on Endothelial Cell Viability

**Figure 1:**
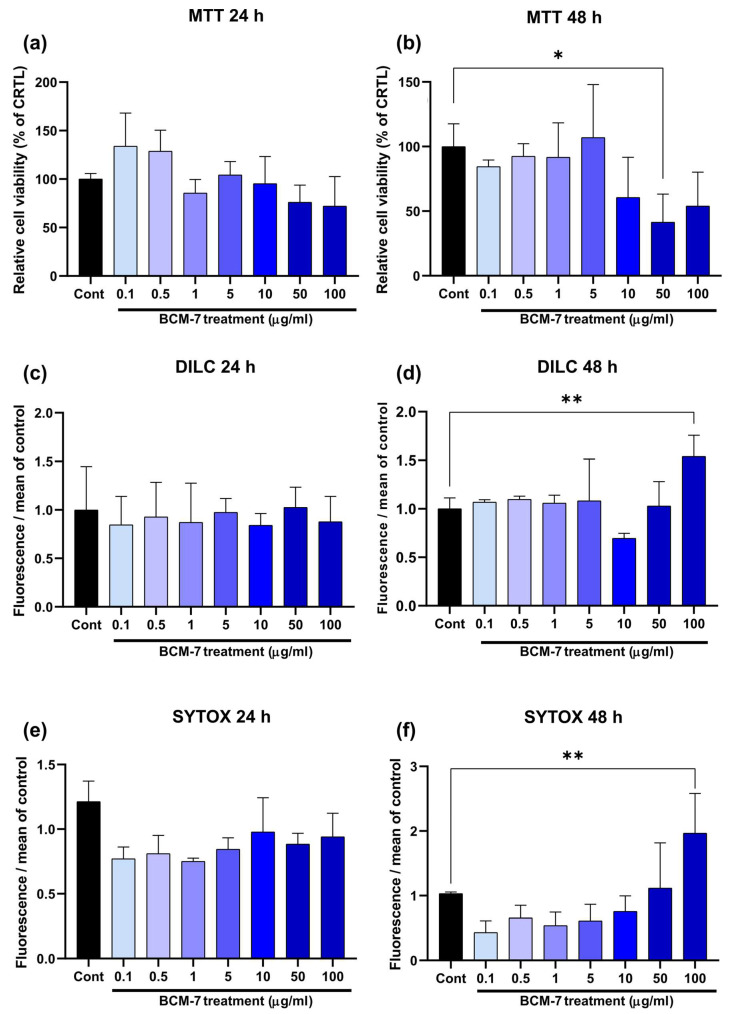
MTT viability assay of HUVECs was evaluated at (**a**) 24 h and (**b**) 48 h. Results are expressed as percentages of the control (100%). Fluorescent DILC1(5) labeling was performed to detect apoptotic cell death under 24 (**c**) and 48 (**d**) h treatment. Necrotic cell process was also determined using SYTOX Green labeling for 24 (**e**) and 48 (**f**) hour time windows. Results show normalized fluorescence values to the control. In the case of the MTT, DILC1(5), and Sytox green assays, we performed three individual experiments with similar results. Data are expressed as the mean ± SD of 4 technical replicates (*n* = 4) from one chosen individual experiment. For statistical analyses, ordinary one-way Anova with Dunnett’s multiple comparison was applied. Asterisks indicate statistical significance: *, *p* ≤ 0.05; **, *p* ≤ 0.01; MTT (3-(4,5-dimetiltiazol-2-il)-2,5-difeniltetrazólium-bromid), DILC, DILC1(5) (1,1′,3,3,3′,3′-Hexamethylindodicarbocyanine iodide), SYTOX (SYTOX™ Green nucleic acid stain), Cont (control), BCM-7 (β-casomorphin-7).

We studied the effects of BCM-7 on endothelial cell viability. HUVECs were exposed to BCM-7 at different concentrations (0.1–100 µg/mL) for 24 and 48 h. We found that the concentrations from 0.1 to 100 µg/mL BCM-7 did not decrease the viability of HUVECs in the 24 h time window (Figure 1a). A similar response was obtained incubating HUVECs with BCM-7 for 48 h; only in the case of 50 µg/mL (57% ± 34) treatment was there an observed significant decrease in cell viability compared to the control group (Figure 1b). Notably, in the case of the 100 µg/mL BCM-7 treatment, no significant cell death was observed relative to the control at 48 h. Considering that the concentrated application of BCM-7 has cytotoxic effects, it may be asked whether lower concentrations also trigger negative cellular processes that cannot be detected by MTT assays. Furthermore, BCM-7 exerts these cytotoxic effects by inducing apoptosis or necrosis. In order to answer these questions, we used DilC1(5) and SYTOX Green fluorescent labeling. The DilC1(5) was used to measure mitochondrial membrane potential in apoptotic cells with a loss in membrane potential reflected in a loss in fluorescence signal in the infrared channel. Thus, we performed dose-response experiments using BCM-7 concentrations ranging from 0.1 to 100 µg/mL. The results show that BCM-7, in line with MTT assay results, did not significantly alter the apoptotic process in either the 24 (Figure 1c) and 48 h (Figure 1b) time windows. Our experiments also investigated whether BCM-7 induces necrosis in HUVECs (Figure 1e,f). We found that BCM-7 did not significantly alter the HUVEC necrotic process in a 24 h treatment (Figure 1e). The incubation of HUVECs with BCM-7 for 48 h resulted in necrosis in cells at the concentration of 100 µg/mL (1.96 ± 0.61) (Figure 1f). BCM-7 can be used without the risk of any biologically relevant cytotoxic actions in this concentration range (≤5 µg/mL). Based on these preliminary data, the concentration of 5 µg/mL BCM-7 was selected for further experiments (except for the intracellular ROS experiment).

### 3.2. BCM-7 Treatment-Induced Intracellular ROS Production in HUVECs

**Figure 2:**
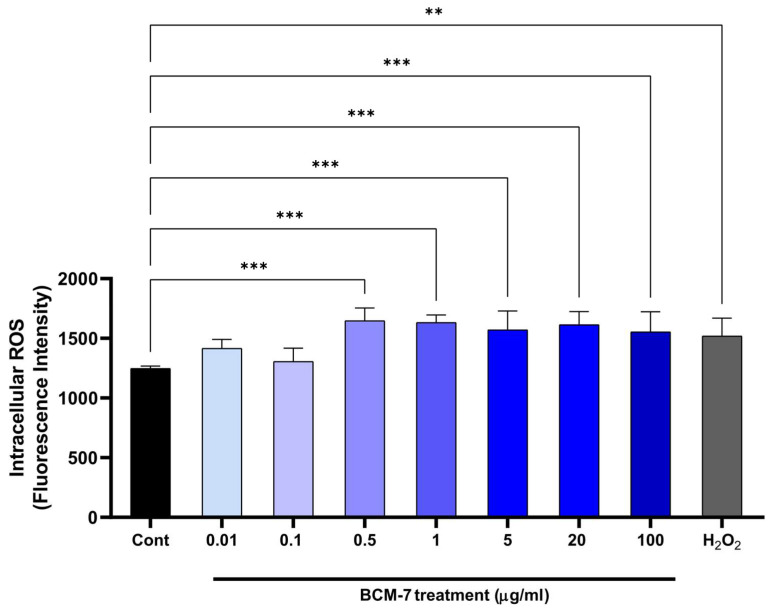
The effect of BCM-7 on the ROS production of HUVECs. HUVECs were treated with BCM-7 (0.01–100 μg/mL) for one hour. As a positive control we used H_2_O_2_ at a 100 µMol concentration. Data are expressed as the mean ± SD. We performed three individual experiments, with four technical replicates (*n* = 4) in every case. The figure represents one individual experiment; two additional experiments yielded similar results. For statistical analyses, ordinary one-way Anova with Dunnett’s multiple comparison was applied. Asterisks indicate statistical significance: **, *p* ≤ 0.01; ***, *p* ≤ 0.001. Cont (control), ROS (reactive oxygen species), H_2_O_2_ (hydrogen-peroxide), BCM-7 (β-casomorphin-7).

To explore whether the unfettered production of ROS is implicated in HUVECs under BCM-7 treatment, we measured ROS production. Compared to the control, BCM-7 significantly increased ROS production at 0.5, 1, 5, 20, and 100 µg/mL concentrations (Figure 2). Slight but not significant differences were observed in the cases of 0.01 and 0.1 µg/mL concentrations. For our experiments we used H_2_O_2_ at a 100 µMol concentration for positive control. As Figure 2 shows, H_2_O_2_ significantly enhanced the intracellular ROS production in comparison to control.

### 3.3. LPS-Induced Proinflammatory Cytokine Expression Was Increased by BCM-7 in Endothelial Cells

**Figure 3:**
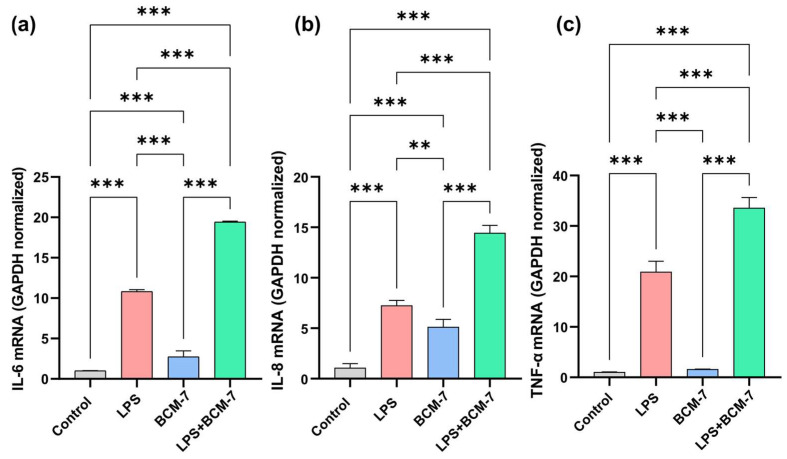
The effects of BCM-7 treatment on LPS-induced, proinflammatory cytokine expression levels. HUVEC/TERT2s were treated with LPS (100 ng/mL) and BCM-7 (5 µg/mL) alone and in combination with LPS (100 ng/mL) for 4 h. Quantitative real-time PCR was performed to assess the gene expression level of IL-6 (**a**), IL-8 (**b**), and TNF-α (**c**). Three individual experiments were performed. Results are presented as the mean ± SD of one independent experiment performed in triplicate (*n* = 3). For statistical analyses, ordinary one-way Anova with Dunnett’s multiple comparison was applied. Asterisks indicate statistical significance: **, *p* ≤ 0.01; ***, *p* ≤ 0.001. Data shown as normalized relative gene expression (normalized to GAPDH) calculated with Livak’ formula. Data shown as mean ± SD. Glyceraldehyde 3-phosphate dehydrogenase (GAPDH), IL-6 (interleukin-6), IL-8 (interleukin-8), TNF-α (tumor necrosis factor-alpha), LPS (lipopolysaccharide) BCM-7 (β-casomorphin-7), LPS + BCM-7 (lipopolysaccharide+ β-casomorphin-7).

To examine the effect of the BCM-7 on proinflammatory cytokine expression in endothelial cells, HUVECs were treated with BCM-7 alone and in combination with LPS for 4 h (Figure 3). Quantitative real-time PCR reactions were performed to assess the expression levels of interleukin-6 (IL-6), interleukin-8 (IL-8), and (tumor necrosis factor-alpha) TNF-α proinflammatory cytokines. As shown in Figure 3a. LPS enhanced the expression of IL-6 compared to the control group. In the case of BCM-7, a moderate increase was found in relation to the control groups. Furthermore, BCM-7 treatment in combination with LPS was able to elevate the observed increase in IL-6 (Figure 3a). In comparison with the control group, LPS significantly elevated the IL-8 proinflammatory cytokine levels (Figure 3b). In line with the IL-6 results, BCM-7 treatment induced IL-8 proinflammatory cytokine expression. Moreover, BCM-7 was able to enhance the LPS-induced IL-8 cytokine expression. In the case of TNF-α, BCM-7 treatment did not result in increased cytokine expression; however, it successfully enhanced the LPS-induced elevation of TNFα levels (Figure 3c). The relative mRNA expression of the investigated TNFα significantly increased as a result of LPS treatment compared to the control group. BCM-7 treatment alone did not significantly influence the changes in the expression of the tested genes. BCM-7 treatment was able to increase LPS-induced TNF-alpha mRNA levels. (Figure 3c).

### 3.4. BCM-7 Remarkably Influences the Expression of Genes That Play an Important Role in Maintaining Endothelial Cell Homeostasis

**Figure 4:**
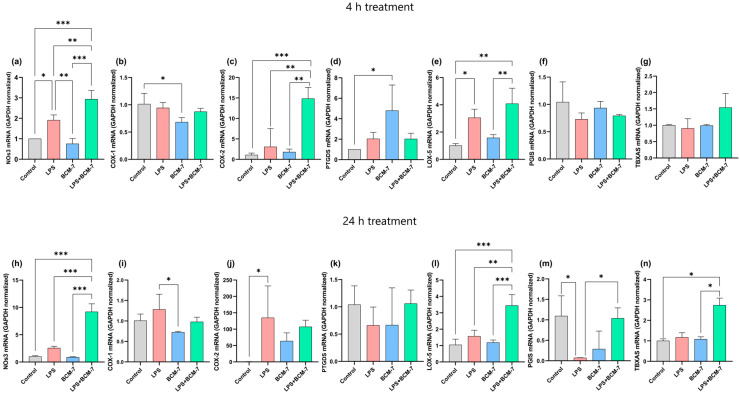
The effects of BCM-7 treatment on HUVEC physiological status. Quantitative real-time PCR was performed to assess the gene expression level of NOS3 (**a**), COX-1 (**b**), COX-2 (**c**), PTGDS (**d**), LOX-5 (**e**), PGIS (**f**), and TBXAS (**g**) after 4 h, and NOS3 (**h**), COX-1 (**i**), COX-2 (**j**), PTGDS (**k**), LOX-5 (**l**), PGIS (**m**), TBXAS (**n**) after 24 h. Data shown as normalized relative gene expression (normalized to GAPDH) calculated with Livak’ formula. Data are expressed as the mean ± SD of three individual experiments with three technical replicates. For statistical analyses, ordinary one-way Anova with Dunnett’s multiple comparison was applied. Asterisks indicate statistical significance: *, *p* ≤  0.05; **, *p* ≤ 0.01; ***, *p* ≤ 0.001. NOS3 (nitric oxide synthase 3), COX-1 (cyclooxygenase-1), COX-2 (cyclooxygenase-2), PTGDS (prostaglandin D2 synthase), LOX-5 (lipoxygenase-5), PGIS (prostaglandin I2), TBXAS (thromboxane A synthase) LPS (lipopolysaccharide), BCM-7 (β-casomorphin-7), LPS + BCM-7 (lipopolysaccharide + β-casomorphin-7).

We aimed to target those genes that play an important role in maintaining endothelial cell homeostasis (Figure 4). For this, HUVECs were exposed with BCM-7 alone and in combination with LPS for 4 and 24 h. The relative transcriptional levels of nitric oxide synthase 3 (NOS3), and expressions of cyclooxygenase-1 (COX-1), cyclooxygenase-2 (COX-2), prostaglandin D2 synthase (PTGDS), lipoxygenase-5 (LOX-5), prostaglandin I2 (PGIS), and thromboxane A synthase (TBXAS) were investigated. Interestingly, in 4 h (Figure 4a) as well as 24 h (Figure 4h) cell treatments, LPS increased the NOS3 mRNA expression levels compared to the control samples. Furthermore, BCM-7 in combination with LPS treatment was able to enhance the aforementioned NOS3 expression in both the 4 (Figure 4a) and 24 (Figure 4h) hour time frames. According to our results, BCM-7 treatment significantly decreased the expression of COX-1 compared to the control groups in a four-hour time window (Figure 4b). After 24 h, decreased expression of COX-1 was observed in the case of BCM-7 in relation to the LPS treatments (Figure 4i). We also tested the relative gene expression level of COX-2 after 4 h (Figure 4c) and 24 h (Figure 4j) cell treatment. BCM-7 in combination with LPS significantly increased the expression of COX-2 after the four-hour treatment. In the case of the 24 h treatment, significant differences were only observed with the LPS treatment compared to the control group. PTGDS mRNA expression was also investigated. In relation to the control group, increased expression was noticed in the four-hour treatment with BCM-7 (Figure 4d). This difference was diminished after 24 h (Figure 4k). LPS increased the mRNA level of LOX-5 after 4 h treatment (Figure 4e). BCM-7 in combination with LPS increased LOX-5 expression in both the 4- (Figure 4e) and 24- (Figure 4l) hour treatments. After 4 h of treatment, no significant change was observed in the PGIS relative gene expression levels with the applied treatment (Figure 4f). Both LPS and BCM-7 treatment reduced the relative gene expression levels of PGIS after 24 h. This significant reduction was not observed with LPS treatment in combination with BCM-7 (Figure 4m). The TBXAS expression level was increased by BCM-7 treatment in combination with LPS after 24 h (Figure 4n).

## 4. Discussion

The present study revealed novel and previously underexplored aspects of the effects of β-casomorphin-7 (BCM-7) on endothelial cells, in particular on the regulation of oxidative stress, inflammatory responses, and enzymes concerned in inflammation and vascular function. We hypothesized that BCM-7 plays a significant role in the development of endothelial dysfunction. The mechanism of action of A1 β-casein on endothelial cell homeostasis is currently incompletely understood; to our knowledge, this is the first study to investigate aspects of these protein fractions in relation to endothelial dysfunction. At the beginning of the experimental series, we determined the optimal concentration range for BCM-7 application. To assess cytotoxic effects, cell viability (MTT), apoptosis, and necrosis assays were performed (Figure 1). Here, we observed that BCM-7 at a 50 µg/mL concentration induced significant cell death at 48 h treatment. However, under the same treatment conditions, no significant cell death was noticed with a concentration of 100 µg/mL BCM-7 treatment. It is also possible that HUVECs activate adaptive or protective mechanisms in response to higher peptide exposure, such as stress response pathways or compensatory signaling, which may mitigate the observed cytotoxicity. Our results showed that BCM-7 at a concentration of 5 µg/mL did not induce significant cytotoxicity. Significant apoptotic and necrotic activity was noticed at 100 µg/mL following 48 h of exposure (*p* ≤ 0.001).

The results of the ROS assay indicate that BCM-7 alone is capable of significantly enhancing oxidative and inflammatory activity in endothelial cells. Maintaining endothelial cell homeostasis is crucial for the preservation of vascular integrity. Oxidative stress is well known to be one of the most important and earliest triggers of endothelial dysfunction [31], which may contribute to long-term vascular wall integrity breakdown, vascular damage [31], increased vascular wall permeability, progression of vascular inflammation and atherosclerotic plaque formation. Our results show that BCM-7 dose-dependently enhanced ROS production (Figure 2), suggesting that BCM-7 alone can induce an intracellular oxidative environment that may contribute to endothelial cell damage. Based on these preliminary data, the concentration of 5 µg/mL BCM-7 was selected for further experiments. The choice of the 5 µg/mL concentration was justified by the results of a previous study by Gard F. 2024 [32]. They investigated the effects of 1% pre-digested A1 milk on the proliferation of human peripheral blood mononuclear cells. The concentration in this product was used to determine the BCM-7 concentration: 5.2 µg/mL.

When we examined the expression of proinflammatory cytokines (IL-6, IL-8, and TNF-α) in mRNA levels, we detected a significant proinflammatory effect of BCM-7, especially for IL-6 and IL-8. To the best of our knowledge, there is no available information about the effect of BCM-7 on the proinflammatory cytokine production of endothelial cells, including the expression of IL-6 and IL-8. However, studies on intestinal epithelial cells have shown that BCM-7 enhances cytokine secretion. [33,34]. The elevation of IL-8 levels may be of particular relevance, as the previous literature identifies IL-8 as a potential biomarker of coronary artery disease (CAD) [35,36,37]. Combined exposure to LPS further increased the gene expression of the cytokines tested, raising the possibility that the inflammatory potential of BCM-7 may be even more significant in the presence of LPS (Figure 3). These cytokines promote the increased adhesion of leukocytes to the endothelium via the inflammatory activation of endothelial cells, increase vascular wall permeability, sustain inflammatory processes, and contribute to a reduction in plaque stability, thereby promoting the progression of atherosclerotic lesions, which may underlie the development of vascular complications [38,39,40,41].

During the investigation of endothelial cell homeostasis, we observed further significant differences. A 24-h exposure to LPS induced COX-2 gene expression, which can be interpreted as part of the inflammatory response. In treatments combined with LPS, an additional increase in COX-2 mRNA expression was already detectable within 4 h. In parallel, a reduction in COX-1 gene expression was also observed, indicating a shift in the balance toward a proinflammatory state.

Thus, increased COX-2 transcription can potentially alter the balance of prostanoid mediator synthesis, thereby promoting the development of an inflammatory environment [42,43]. LPS treatment induced the expression of the LOX-5 gene, suggesting that this enzyme is produced at increased levels during the inflammatory response.

Exposure combined with LPS further increased LOX-5 mRNA expression at the 4 and 24 h treatment time points. The increase in LOX-5 in mRNA levels suggests that alternative pathways of arachidonic acid metabolism that promote inflammation are also activated. This suggests that BCM-7 may exert a regulatory effect on the gene expression components of inflammation-related lipid mediator pathways [44].

An increase in NOS3 (eNOS) gene expression was observed with LPS and combined treatment. This change in eNOS expression may be interpreted as a compensatory mechanism to maintain endothelial survival in the face of oxidative stress and inflammation, but paradoxically, this increased eNOS activity may also contribute to the phenomenon of “eNOS uncoupling”, which may further increase ROS production [45,46]. Based on our results, BCM-7 influences the regulation of arachidonic acid metabolism gene expression at multiple levels, which may indirectly promote the activation of inflammatory and thrombotic processes. The increase in PTGDS gene expression induced by BCM-7 is also an interesting observation at 4 h of treatment, as the increase in prostaglandin D_2_ synthesis may be involved in the regulation of inflammatory and thrombotic processes. In contrast, the changes in PGIS and TBXAS expressions were less pronounced, although an increase in TBXAS (Figure 4) mRNA levels over 24 h combined exposure may already indicate the subsequent activation of the thromboxane pathway; i.e., it may enhance the development of a protrombotic state [47,48].

Our study has several limitations. First, based on the viability assay and previous research conducted with BCM-7 on cell models, we applied the 5 µg/mL concentration of BCM-7 for cell treatment. For viability and the ROS measure we also tested BCM-7 in lower concentrations, including 0.1, 0.5 and 1 µg/mL. However, for the gene and protein expression experiment we only used BCM-7 in a 5 µg/mL concentration. In our opinion, the use of lower BCM-7 concentrations (e.g., 1 µg/mL or less) on the HUVEC cell line is less than ideal for several reasons. When lower concentrations of BCM-7 are used, the amount of peptides does not reach the level required for meaningful stimulation of endothelial cells, so the biological effect may remain weak or undetectable. In addition, the measurable response is small compared to the background noise, which reduces statistical power and makes it difficult to interpret the experimental results. Therefore, we did not work with lower doses, even if they did not cause apoptosis, necrosis, or cytotoxicity. Second, some discrepancies were also noticed during our experiment, such as that BCM-7 at 50 µg/mL concentration induced significant cell death at 48 h treatment, but not in 100 µg/mL concentration. The mechanism which causes this phenomenon is unknown. One possible mechanism is the activation of adaptive or protective mechanisms, such as heme oxygenase activation in response to a higher concentration of BCM-7. Furthermore, our research did not address the study of adaptive mechanisms that respond to endothelial stress, such as heme oxygenase activation and GSH measurement, i.e., redox homeostasis. We believe that it may be important to study the effect of BCM-7 on endothelial cell redox homeostasis, but this was not the subject of our current research. We used the immortalized HUVEC/TERT2 cell line as a model in our research. Moreover, we did not determine the functional end products of the COX/LOX pathway (PGE_2_, PGD_2_, LTB_4_, 5-HETE), therefore the results are limited to activation at the transcriptional and signaling levels. Quantification of lipid mediators and the use of COX/LOX inhibitors will be the subject of our future research.

Overall, our findings indicate that BCM-7 possesses significant proinflammatory and oxidative stress-inducing potential in endothelial cells at transcription levels, which may contribute to endothelial dysfunction, atherosclerosis, and an increased risk of thrombosis. Our results raise the possibility that BCM-7, particularly under conditions of chronic exposure, may act as a direct risk factor in the development of cardiovascular diseases, especially in inflammatory pathologies associated with endothelial damage. It is important to note that the precise physiological role of milk-derived BCM-7 remains the subject of ongoing intensive research. The inflammatory and oxidative effects we observed may be of particular clinical relevance in those with or associated with leaky gut syndrome or at increased cardiovascular risk. Further research will be essential to clarify what functional lipid mediator changes and vascular effects are associated with the transcriptional changes induced by BCM-7 in vivo, and their potential clinical relevance for both cardiovascular prevention and dietary recommendations.

## 5. Conclusions

Our study was the first to describe in detail the complex effects of β-casomorphin-7 (BCM-7) on human endothelial cells, revealing its possible role in the regulation of signaling and transcriptional processes associated with endothelial dysfunction. We first demonstrated that BCM-7 enhances the gene expression of oxidative stress markers and inflammatory cytokines (IL-6, TNF-α, IL-8). Furthermore, BCM-7 influences the transcriptional activity of inflammatory pathways associated with COX-1, COX-2, PTGDS, LOX5, NOS3, PGIS, and TBXAS enzymes in the HUVEC/TERT2 cell line, especially in the presence of LPS. These observations suggest that BCM-7, as a biologically active peptide derived from milk protein, may contribute to the regulation of the endothelial inflammatory response and thus play a role in the development of endothelial dysfunction. Further in vivo studies are needed to clarify how BCM-7-induced transcriptional and signaling changes affect vascular homeostasis and the risk of cardiovascular disease in the long term.

## Data Availability

The data that support the findings of this study are available on request from the corresponding author.
